# Causal relationships between diseases mined from the literature improve the use of polygenic risk scores

**DOI:** 10.1093/bioinformatics/btae639

**Published:** 2024-10-26

**Authors:** Sumyyah Toonsi, Iris Ivy Gauran, Hernando Ombao, Paul N Schofield, Robert Hoehndorf

**Affiliations:** Computer, Electrical and Mathematical Sciences & Engineering, King Abdullah University of Science and Technology, Thuwal 23955, Saudi Arabia; Computer, Electrical and Mathematical Sciences & Engineering, King Abdullah University of Science and Technology, Thuwal 23955, Saudi Arabia; Computer, Electrical and Mathematical Sciences & Engineering, King Abdullah University of Science and Technology, Thuwal 23955, Saudi Arabia; Department of Physiology, Development & Neuroscience, University of Cambridge, Cambridge CB2 3EG, United Kingdom; Computer, Electrical and Mathematical Sciences & Engineering, King Abdullah University of Science and Technology, Thuwal 23955, Saudi Arabia; SDAIA–KAUST Center of Excellence in Data Science and Artificial Intelligence, King Abdullah University of Science and Technology, Thuwal 23955, Saudi Arabia; KAUST Center of Excellence for Smart Health (KCSH), King Abdullah University of Science and Technology, Thuwal 23955, Saudi Arabia; KAUST Center of Excellence for Generative AI, King Abdullah University of Science and Technology, Thuwal 23955, Saudi Arabia

## Abstract

**Motivation:**

Identifying causal relations between diseases allows for the study of shared pathways, biological mechanisms, and inter-disease risks. Such causal relations can facilitate the identification of potential disease precursors and candidates for drug re-purposing. However, computational methods often lack access to these causal relations. Few approaches have been developed to automatically extract causal relationships between diseases from unstructured text, but they are often only focused on a small number of diseases, lack validation of the extracted causal relations, or do not make their data available.

**Results:**

We automatically mined statements asserting a causal relation between diseases from the scientific literature by leveraging lexical patterns. Following automated mining of causal relations, we mapped the diseases to the International Classification of Diseases (ICD) identifiers to allow the direct application to clinical data. We provide quantitative and qualitative measures to evaluate the mined causal relations and compare to UK Biobank diagnosis data as a completely independent data source. The validated causal associations were used to create a directed acyclic graph that can be used by causal inference frameworks. We demonstrate the utility of our causal network by performing causal inference using the do-calculus, using relations within the graph to construct and improve polygenic risk scores, and disentangle the pleiotropic effects of variants.

**Availability and implementation:**

The data are available through https://github.com/bio-ontology-research-group/causal-relations-between-diseases.

## 1 Introduction

Many complex diseases occur in clusters where a primary disease is preceded or accompanied by other diseases or symptoms, i.e. they form co-morbidities. The relationships between co-morbid diseases can provide insights into common underlying causes, shared genetic pathways, and pathobiology. The relationship between co-morbid diseases is complex and is generally established by statistical approaches to disease co-prevalence in a population of patients, or by the construction of knowledge graphs whose disease or phenotype connectivity is established though observation of prevalence in the relevant population (Hidalgo *et al.* 2009, Klimek *et al.* 2015). In some cases, co-morbid diseases may have a common underlying cause, or there may be a direct or indirect causal relationship between them insofar as one disease is effectively a sequel of the previous one and caused by pathological changes initiated by the primary disease (Rovetto and Mizoguchi 2015). An example of such a chain may be found in the causal sequence of type 2 diabetes → hyperglycemia → microvascular disease → diabetic retinopathy (Cheung *et al.* 2010) which is dependent on the time course and severity of the primary disease.

Establishing causal relations between diseases is a significant challenge in epidemiology because randomized controlled trials, the gold standard for establishing causality, cannot be applied. Instead, cohort or case/control studies are used and statistical methods employed to control for confounding variables such as genetic pleiotropy, population structure, and the environment. Additionally, models such as the Bradford Hill criteria (Hill 1965) can be used to assess evidence for a causal relation between two diseases.

Many discoveries and hypotheses about causal relations are communicated through natural language in the scientific literature. These assertions are not directly accessible for many downstream analytical tasks because they are delivered in unstructured text formats. Previous studies have attempted to extract assertions of causal relations from text using Natural Language Processing. For example, different causal patterns can be used to extract causal disease relations from PubMed article abstracts (Lee and Shin 2017). More recently, Long Short-Term Memory networks were utilized to extract dependency patterns between cause–effect pairs (Kabir *et al.* 2022). Another study extracted causal relations between diseases from the UK National Health Service (NHS) data resource and provided the data in the Resource Description Format (RDF) (Yu and Reiff-Marganiec 2022). Several efforts have attempted to extract causal relations between diseases on a large scale using other data sources. For instance, a disease association network is used where the edges between highly associated diseases are oriented using metabolic pathway data (Bang *et al.* 2016). More recently, a knowledge graph was constructed by leveraging Large Language Models (LLMs) to answer causal questions about diseases (Arsenyan *et al.* 2024). Similarly, LLMs were used to effectively address causal questions (Zhang *et al.* 2023).

There are several limitations in the existing approaches that rely on extracted disease–disease causal relations. Firstly, most studies are limited to a small set of selected diseases which do not fully capture the complexity of disease interactions. Secondly, the extracted causal relations are not mapped to identifiers used to characterize diseases, limiting their interoperability and therefore their integration into computational methods. Thirdly, the relations are not supported by any quantitative or qualitative measures and are generally limited to a single source of data.

We have developed a resource of causal relations between diseases; each disease is mapped to a common identifier and each assertion of a causal relation is supported by quantitative data. In particular, we collected relations that describe directed pairs of the following format: “Disease x can cause the onset of disease y” (x→y). To identify assertions of causality between diseases on a large scale, we extracted relations mentioned in abstracts of literature articles using lexical patterns to extract candidate pairs. Subsequently, we linked the diseases to identifiers of the International Classification of Diseases, tenth revision, Clinical Modification (ICD-10-CM) (Steindel 2010). We provide quantitative and qualitative data for each relation: number of annotations, response about the relation from an LLM [GPT-4 (OpenAI 2023)], correlation coefficient, test for independence, and temporal sequence of dates of diagnosis.

We extract a sub-network of causal relations that forms a Directed Acyclic Graph (DAG) and can be used as a Structural Causal Model (SCM). This framework, developed extensively by Judea Pearl (Pearl 2009), is fundamental to distinguishing between correlation and causation in causal inference. To demonstrate the utility of the DAG and evaluate it further, we apply it to the construction of Polygenic Risk Scores (PRSs) which are widely used to estimate individual-level risk for traits or diseases by summing the effects of multiple genetic variants across the genome (Dudbridge 2016). The effects of variants are commonly estimated using observational genetic data from individuals exhibiting the trait of interest. Methods to construct PRSs can use priors like biological functions to select better variants (Xiang *et al.* 2024). One of the biological priors used is the association among multiple traits, which has been shown to improve the signal of some PRSs by estimating the effects of genetic variants based on multiple traits (Zhai *et al.* 2023). In this case, causative relations between diseases can be leveraged to choose the diseases to be included in such multi-trait PRS construction. We demonstrate that a multi-trait PRS for an outcome can be constructed by aggregating its PRS with those of its causative diseases. Furthermore, we show that it is possible to compute scores for diseases without available PRSs by utilizing information about their causative diseases.

Disentangling the patterns of genetic pleiotropy, where a single variant affects multiple phenotypes or diseases, is challenging using association studies alone (Watanabe *et al.* 2019). Knowledge of causative relationships allows discrimination between the situation where one set of diseases is part of a causative chain originating in a single gene (vertical or mediated pleiotropy), or where the gene in question independently affects common pathways or disease modules of which it is a member (horizontal pleiotropy) (Oti and Brunner 2006, Solovieff *et al.* 2013, Watanabe *et al.* 2019). We further show how to use the causal graph we developed to disentangle pleiotropic effects of variants identified in association studies.

## 2 Materials and methods

### 2.1 Mining causal relations from text

We used the PubMed https://pubmed.ncbi.nlm.nih.gov/ baseline data (NCBI 1996), as a literature resource to mine causal relations. First, we created a dictionary of ICD-10-CM names (Steindel 2010) via the Unified Medical Language System (UMLS) (Bodenreider 2004) which covers 345 115 names corresponding to 3960 ICD-10-CM codes. However, because ICD-10-CM covers a wide range of health-associated concepts, over and above diseases and phenotypes (e.g. the code V89 refers to “Motor- or nonmotor-vehicle accident, type of vehicle unspecified”), we restricted ourselves to a subset of ICD-10-CM codes that are mapped to the Disease Ontology (DO) ([Bibr btae639-B45][Bibr btae639-B44]). We used the mappings from DO classes to ICD-10-CM codes found in the DO (i.e. the mappings that are included as annotation properties, or xref statements, in the DO distribution files) which resulted in 2457 ICD-10-CM codes. We filtered using the three-character category and ended up with a dictionary of 85 881 names covering 2968 codes which we applied on the abstracts to identify disease mentions. Mappings between DO and ICD-10-CM as well as other terminologies were created by the DO curation team, based on semi-automated mappings combined with expert curation (Schriml *et al.* 2018, Baron *et al.* 2023).

From the corpus, we retrieved all sentences where two diseases co-occur. Then, we used lexical causal patterns provided in a previous work (Lee and Shin 2017) as well as additional patterns (see Supplementary Table S4 for all used patterns) to extract pairs of diseases where a statement claims that one disease causes the other. To filter out spurious relations, we excluded relations between diseases that share the same three-character category in ICD-10-CM. This yielded 16 808 sentences covering 8191 unique relations.

### 2.2 Enriching relations and creating a directed acyclic graph

To further support the extracted relations, we generated several measures using the UK Biobank (UKB) (Sudlow *et al.* 2015) dataset. The UKB is a prospective study which originally included around 500 000 participants aged 40–69. The UKB provides a wide range of reported diagnoses of individuals using ICD-10-CM. For each cause–effect disease pair in our dataset, we calculated the following measures between the cause and outcome diseases on the UKB data:

- Phi correlation coefficient (ϕ): Given some causative disease x and outcome disease y, ϕ is calculated as follows:
  $$\phi =\frac{n_{11}n_{00}-n_{10}n_{01}}{\sqrt{n_{1\cdot }n_{0\cdot }n_{\cdot 1}n_{\cdot 0}}}$$

Here, $n_{11}$, $n_{00}$, $n_{10}$, and $n_{01}$ represent the number of individuals who have both diseases, have neither x nor y, have x but not y, and have y but not x, respectively. Similarly, $n_{1\cdot }$, $n_{0\cdot }$, $n_{\cdot 1}$, and $n_{\cdot 0}$ represent the total number of individuals who have x, do not have x, have y, and do not have y, respectively. If there were no samples for either diseases, we set the value of this measure to 0.

- Dependence (dep): Determined via a Chi-square $\left(\chi^{2}\right)$ test for independence. Since diseases can be involved in multiple relations, we adjusted the *P*-values using the Benjamini-Hochberg method with a threshold of 0.05. We coded this measure as 1 if the null hypothesis is rejected and 0 otherwise.
- Temporal correspondence of diagnosis date ($D_{\mathrm{date}}$): Given some causative disease x and outcome disease y, we calculated this measure as follows:
  $$\frac{\sum_{i=1}^{N}I(d_{x}^{i}\leq d_{y}^{i})}{N}$$

Here, N represents the total number of individuals exhibiting both diseases. $d_{x}^{i}$ and $d_{y}^{i}$ represent the year at which the $i^{\text{th}}$ individual was diagnosed with x and y, respectively. I(·) is the indicator function which equals 1 if the condition inside the parentheses is true, and 0 otherwise. The dates of diagnosis were retrieved using UKB data-field 41280. If there were no samples exhibiting both diseases, we set this measure to 0.

Additionally, we compute two further measures for each disease pair:

- Number of annotations found from text ($n_{\mathrm{annot}}$): This corresponds to the number of sentences that support the relation. This measure is set to 1 if the number of sentences supporting the relation is greater than the median (which is 1) and 0 otherwise.
- Large Language Model confirmation (GPT): We asked GPT-4 whether the causative disease can cause the outcome disease and to reply with “Yes” or “No” only (see Supplementary Table S5 for the details on the prompt we used). This is coded as 1 if it responds with “Yes” and 0 otherwise.

We used these measures to create a combined score that ranges between 0 and 1 for each causative disease pair. The score can be interpreted as a confidence in whether a causal relation holds between the two diseases. Therefore, we assigned a score to each relation by equally weighing the measures as follows:

(2) $$\mathrm{score}=\frac{\phi +\mathrm{dep}+D_{\mathrm{date}}+n_{\mathrm{annot}}+\mathrm{GPT}}{5}$$

We also used this *score* to create a DAG from the total set of extracted disease pairs. In the DAG, each unique ICD-10-CM code is represented as a node and the causal relations are directed edges. We sorted the edges by their score and incrementally added the edges. Whenever a cycle was formed, the edge with the lowest score in the cycle was removed. A total of 602 edges were removed in the process, resulting in a DAG with 1860 nodes and 7589 edges.

### 2.3 Calculation of polygenic risk scores

We utilized the UK Biobank (UKB) data to assess the improvement of some PRSs using the mined causal relations. Initially, we conducted quality control on the samples following the protocol outlined in Khera *et al.* (2018). Briefly, we excluded samples with high heterozygosity or genotype missing rates and discrepancies between reported and genetic sex. Additionally, we filtered out first-degree relatives based on the kinship coefficient provided by UKB (data field 22012). We further restricted our analysis to white British individuals which are the majority in UKB, resulting in 425 573 individuals.

As examples, we retrieved pre-calculated PRSs from the Polygenic Score (PGS) catalog (Lambert *et al.* 2021). The scores for the causative diseases were chosen such that they were fitted on a European cohort but not on the UKB itself. We retrieved the PRSs of the top three causes (due to data availability), using the three-character code of the causes. We utilized Plink (Chang *et al.* 2015) to automatically compute the scores on the imputed genotype data from UKB, using harmonized scores provided by the PGS catalog. We removed duplicated variants and excluded variants with low imputation quality as well as ambiguous variants. Subsequently, we reported the ROC AUC performance on a random split of 90% of the UKB samples, with the remaining 10% used for parameter fitting of logistic regression.

For the large-scale analysis, we used Plink to obtain GWAS results for 149 diseases (see Automatic polygenic score calculation in Supplementary Materials for further details). To fit the PRSs, we used PRSice (Choi and O’Reilly 2019) which is designed to efficiently calculate PRSs on a large scale. Using the GWASs from the first step, we used the default parameters of PRSice to fit 149 PRSs. We then evaluated the performance of each of the PRSs. Finally, we combined the PRSs of the outcome and the causes using logistic regression on a held out set.

## 3 Results

### 3.1 Validation of the mined disease–disease causal relations

We used lexical patterns to automatically mine causal relations between diseases from abstracts of biomedical articles. This yielded 16 808 sentences that cover 8191 unique relations. The relations spanned 1860 unique ICD-10-CM codes. Among the relations, 2969 were supported by more than one mention.

To validate the mined relations, we used some of the criteria that indicate a causal relationship defined by Bradford Hill (Hill 1965). We first assessed whether the diseases in the mined relation are associated, and then determine the strength of the association. While association does not imply causation, it is often a useful heuristic before establishing causality, as many causative relations exhibit strong association (Hernán and Robins 2018). To this end, we utilized the ICD-10-CM reported diagnoses of individuals from the UK Biobank (UKB) (Sudlow *et al.* 2015) to measure association. In particular, we calculated ϕ correlation coefficients of the mined disease pairs (see Section 2.2).

To test if our mined relations exhibit a distinct pattern of association, we compared the ϕ coefficient of our mined relations against randomly selected pairs of diseases. The ϕ correlation coefficient can measure the correlation between binary variables (Kalgotra *et al.* 2020). Due to many diseases having a small sample size of cases (see Supplementary Fig. S1), we restricted the pairs to include ICD-10-CM codes which have been observed in at least 1000 individuals in UKB. This resulted in 577 causal pairs for which we sampled an equal number of random pairs. We applied Fisher’s z-transformation on the ϕ coefficients followed by an independent sample *t*-test. We found a statistically significant difference in the mean transformed correlation coefficients between the two groups of relations (mined vs. random) in the UKB diagnostic data (p≪0.001, *t*-test). Additionally, we measured the effect size using Cohen’s d which is calculated by taking the difference of the means of the two groups over the pooled standard deviation. We found Cohen’s d to be 0.86, indicating a large effect size (Muller 1989).

In addition to correlation, if a causal relation exists between two variables, then a statistical dependence should be present. Therefore, we assessed whether the disease pairs in the 8191 relations we mined are statistically dependent. To this end, we used the UKB diagnostic data for individuals to test for independence of disease pairs. To account for multiple comparisons where diseases are involved in several relations, we adjusted the *P*-values using the Benjamini-Hochberg method (α=0.05). We found 2160 disease pairs to be dependent (the null hypothesis of the Chi-square test of independence was rejected) and 2919 to be independent. The remaining pairs of diseases could not be tested due to small sample sizes.

As another validation of our extracted relations, we used the reported date of diagnosis from the UKB data to determine whether the mined relations meet the temporal correspondence criteria of causation (Hill 1965) where the cause occurs before the outcome. To this end, we calculated a ratio of the number of individuals diagnosed with the cause in the same or some prior year to being diagnosed with the outcome, weighted by the number of individuals exhibiting both diseases (see Section 2.2). However, some pairs of diseases do not co-exist in any individual, which limits our analysis to 3274 relations. Supplementary Fig. S2 shows the distribution of the aforementioned ratio for the date of diagnosis of the mined relations. In the figure, most of the relations exhibit a ratio ≥0.5, indicating high temporal dependency for the majority of relations.

To evaluate the accuracy of the lexically-mined relations, we manually curated 100 relations. An expert (P.N.Schofield) was provided with a list of the 1860 diseases that we identified in the literature. Subsequently, we asked the expert to provide 50 directed pairs of diseases for which a consistent (reproducible) and temporal (one disease occurs after the other) association with a plausible biological mechanism for a causal relation exists, based on the expert’s biological and medical background knowledge (Expert curation strategy in Supplementary Materials). We ranked the list of diseases by the number of relations in which they are involved, and asked the expert to provide 50 pairs of diseases for which no relation with the above criteria is known to exist. Using the curated relations, we evaluated the expected accuracy of the complete set of mined relations as 84%.

Lastly, we utilized additional background knowledge in the form of a Large Language Model (GPT-4) (OpenAI 2023) to confirm or negate the 8191 mined relations. GPT-4 confirmed 3687 relations and negated the rest. We queried each relation once, but note variations in GPT-4’s responses; for instance, when querying whether hypertension (I10) can cause acute kidney failure (N17), GPT-4 provided conflicting responses on different occasions.

### 3.2 A directed acyclic graph of causal relations between diseases

Many causal inference methods such as causal Bayesian networks are only applicable to acyclic graphs (Pearl 2009) that represent causal relationships between variables and help to identify potential confounders and the pathways through which causation can flow. By causality, we mean “statistical causality” which is determined by the predictive ability of a candidate variable x for an outcome variable y. A SCM formalizes causal relationships, providing a framework to identify whether variable x can help reduce error in predicting an outcome variable y. An SCM is a useful tool that helps to distinguish between correlation and causation in causal inference.

We organized the mined relations into a directed graph where nodes represent diseases identified by ICD-10-CM codes and edges denote causal relations between them. The graph we mined from literature contains cycles, and SCMs should be acyclic. Our approach to generate a Directed Acyclic Graph (DAG) from this cyclic graph aims to retain the edges with the highest confidence and omits edges with lower confidence. To measure confidence, we assigned a score to each edge in our graph by defining five measures (Table 1), and we combine these five measures into a single combined score (see Section 2.2 and Supplementary Table S1 for further details). To create a DAG, we sorted the edges by their combined scores and sequentially included edges with the highest scores first. Whenever a cycle was formed, we discarded the edge with the lowest score in the cycle.

**Table 1.** The measures used to score relations and their sources.

Source	Measure
Background knowledge	Number of annotations
	GPT-4 confirmation
Patient records	Statistical dependence
	ϕ correlation coefficient
	Correspondence of diagnosis dates

**Figure 1.**
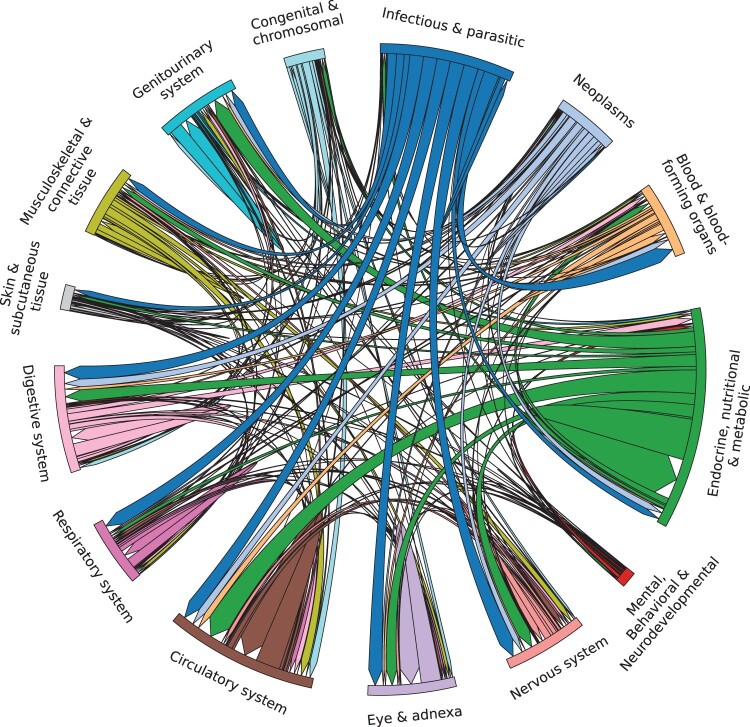
Chord diagram illustrating the causal relations in the DAG grouped by ICD-10-CM chapters. We retained only chapters with more than 100 relations.

Once we removed cycles and generated a DAG from the causal relations, we examined the distribution of the relations in the DAG according to their ICD-10-CM chapters in Fig. 1. We find that “Infectious and parasitic diseases” as well as “Endocrine, nutritional & metabolic diseases” have strong outgoing links to most chapters. As an example, we investigated the five top-scoring relations (coronary atherosclerosis → angina pectoris, coronary atherosclerosis → myocardial infarction, anti-glomerular basement membrane disease → glomerulonephritis, alcoholic liver cirrhosis → portal hypertension, and anorexia nervosa → malnutrition) and find that these relations are well known and supported in literature (Vlodaver *et al.* 1972, Lahmer and Heemann 2012, Libby *et al.* 2019, Iwakiri and Trebicka 2021, Puckett *et al.* 2021, McConnell and Iwakiri 2023).

The DAG can be used for causal inference frameworks. By referring to the structure of the DAG, the do-calculus (Pearl 2009) can be applied to answer certain causal questions. For instance, let us consider the query: “What is the causal effect of the presence of urinary bladder stones (N21.0) on the incidence of vesicorectal fistulas (N32.1)?”. This query can be represented as P(N32.1|do(N21.0=1)), where the do operator denotes intervention on N21.0, with 1 indicating its presence. In our DAG, vesicorectal fistula has a single cause which is urinary bladder stones. Additionally, urinary bladder stones are not linked to any other disease. Other variables can also be incorporated into the DAG such as sex, age, and lifestyle factors. Let us consider age to be the only confounder. In this case, age meets the backdoor criterion (Pearl 2009), allowing us to identify P(N32.1|do(N21.0=1)) using the formula:

$$\sum_{a}P(N32.1|N21.0=1,\text{age}=a)\cdot P(\text{age}=a)$$

We computed the effect of intervention for bladder stones on the development of vesicorectal fistulas from the UKB population on individuals aged 50 and above:

P(N.32=1|N21.0=1,age≥50)=0.0039

The probability is low, which is consistent with the literature as it is rare for urinary bladder stones to cause vesicorectal fistulas (Naguib and Sharaf 2009, Elnaim and Mohamed 2014).

### 3.3 Application to polygenic risk scores

Causal relations also have the potential to improve the development of genetic risk scores because they can identify and leverage common etiology and interdependence of multiple diseases. PRS are designed to determine an individual’s risk for a trait or disease. The PRS of an individual is typically computed as a linear combination of the genotype values ($G_{i}$) of an individual’s genetic variants and the corresponding weights ($w_{i}$) assigned to each variant (Dudbridge 2016). PRSs assume that variants are independent, associated with the outcome, and that each variant (i) contributes a certain weight ($w_{i}$) to the total risk. For n genetic variants, the PRS of an individual can be calculated as follows:

$$\text{PRS}=\sum_{i=1}^{n}\left(w_{i}\cdot G_{i}\right)$$

We utilized relations from the created DAG to generate PRSs and show that our approach can improve the performance (measured by ROC AUC) of some PRSs. In particular, we adopted a Bayesian causal networks framework to compute modified PRSs. To estimate the PRS of a given disease node Y$\left({\text{PRS}}_{Y}\right)$, we condition on its parent nodes (Pa(Y)), representing directly connected causative diseases according to the DAG. Since we often have access to PRSs rather than directly observing the presence or absence of diseases, we assume that we only have access to PRSs. Given a disease Y and its set of directly causative parent diseases (Pa(Y)), we computed a modified PRS as follows:

(1) $${\text{ModifiedPRS}}_{Y}=\sum_{i=1}^{\left|\text{Pa}(Y)\right|}\alpha_{i}\cdot {\text{PRS}}_{\text{Pa}{\left(Y\right)}_{i}}+\beta \cdot {\text{PRS}}_{Y}$$

Here, $\alpha_{i}$ represents the weight assigned to each disease in Pa(Y), indexed by i, and β represents the weight assigned to the PRS of disease Y in the combined risk score. We used logistic regression to fit both parameters on a validation set (see Section 2.3).

As illustrative examples, we obtained previously published PRSs from the PGS catalog (Lambert *et al.* 2021). In particular, we used risk scores for hypertension (Maj *et al.* 2022) (I10), heart failure (I50) (Sinnott-Armstrong *et al.* 2021), coronary heart disease (CHD, I25) (Mars *et al.* 2020), myocardial infarction (I21), and angina pectoris (I20) (Sinnott-Armstrong *et al.* 2021). We applied the published scores (as described in Section 2.3) and denote them as PRS${}_{\mathrm{outcome}}$. To fit the modified score, we used logistic regression on a held out set from the UK Biobank. Table 2 shows the performance for each of the outcome diseases. In all cases, the modified risk score for the outcome disease improved patient identification in the UKB population.

**Table 2.** ROC AUC results comparing the original PRS and the modified PRSs.^a^

Causes	Outcome	**PRS** ${}_{\mathrm{outcome}}$	Modified PRS
Hypertension, CHD, myocardial infarction	Heart failure	0.580	0.678
Hypertension, CHD	Myocardial infarction	0.659	0.779
CHD	Angina pectoris	0.655	0.784

a **Modified PRS** refers to the PRS fitted using the outcome PRS and PRSs of the causes via logistic regression.

To test the relations on a larger scale, we retained relationships from our DAG where both the causative and outcome diseases have 1000 or more cases in UKB. Our analysis included 149 diseases across 471 relationships. We automatically fitted PRSs for all 149 diseases individually (refer to Section 2.3 for further details). As in our earlier approach, we used these PRSs to develop a modified PRS that combines the scores of the causes with the outcome, resulting in 111 modified PRSs. We evaluated the ROC AUC performance of the modified PRSs in comparison to the original outcome’s PRS. Our analysis revealed a small (0.73%) but significant (*P*-value ≈0.00006, Wilcoxon signed-rank test) increase in ROC AUC.

It is also possible to compute a PRS for a disease that either has a small number of samples or does not have an available PRS. In such case, we used the PRSs of the disease’s precursor conditions and consider the weight for the disease’s PRS (β) to be zero. We showed this by calculating the PRS for cardiomegaly (I51.7) and pulmonary edema (J81) using the PRS of hypertension (I10) and myocardial infarction (I21) as retrieved from our DAG (see Supplementary Table S2). Neither cardiomegaly nor pulmonary edema have corresponding PRSs in the PGS catalog.

### 3.4 Causal relations help disentangle pleiotropic effects

Many variants overlap between the PRSs of the chosen diseases; most of the variants of the outcomes are shared with the causes in the PRSs we obtained from the PGS catalog (99% heart failure, 88% myocardial infarction, and 88% for angina pectoris). This is a well-known phenomenon in the literature where genes have pleiotropic effects (Watanabe *et al.* 2019). In such setting, we exhibit chains of the following nature: variant → causative disease → outcome disease. In graphs representing causative relations, chains have properties that we can exploit to disentangle effects of variables. In particular, if an outcome disease is dependent on a variant, and if the outcome is conditionally independent of the variant given the causative disease (outcomedisease⊥⊥variant∣causativedisease), then we can conclude that the effect of the variant on the outcome disease is entirely mediated through the causative disease (Pearl 2009).

As a case study, we obtained significantly associated variants ($p<10^{-8}$) to CHD and angina pectoris from the GWAS Catalog which provides access to summary statistics of published GWAS studies (Sollis *et al.* 2023). We retained variants that are shared between the two diseases resulting in 43 variants. Subsequently, we tested conditional independence using a $\chi^{2}$ test for each variant to test for the following:

angina pectoris⊥⊥variant | CHD

We extracted variants with *P* > 0.05 suggesting conditional independence. We found 30 variants to be conditionally independent of angina pectoris given CHD (see Supplementary Table S3). We queried the mapped genes of the variants (as reported by the GWAS Catalog) for evidence from the Online Mendelian Inheritance in Man (OMIM) which reports curated gene–phenotype relations (Amberger *et al.* 2014). We found two variants in APOE, a gene that is associated with coronary artery disease in OMIM, and a variant in PCSK9, a gene that is linked to familial hypercholesterolemia. Notably, familial hypercholesterolemia is a cause for CHD within our DAG (see Expert validation of the case study in Supplementary Materials).

## 4 Discussion

In order to capture causal chains linking complex diseases, we have used lexical patterns to mine relations between diseases from the literature, and mapped them to ICD-10-CM identifiers. There are caveats to this approach: publication bias, as some diseases and relations might be over- or under-represented in the literature, and false positives and false negatives. False positives can arise from incorrect detection and mapping of diseases and by sentences with complex semantic structures. Conversely, false negatives may result from diseases that were not identified and lexical patterns that we have not covered. However, we can identify additional relations using causal chains from the cause to the outcome according to our network of causal relations. For example, eight of the expert-curated causal relations were not directly asserted in the text-mined data but could be found in the generated graph via directed paths.

To mitigate the effect of false positives and provide further evidence, we used multiple measures based on two main sources of data: background knowledge and empirical data from patient records. The measures aim to score the relation’s strength, temporal consistency, and confidence with respect to background knowledge. Firstly, we measured correlation and tested for independence; secondly, we inspected the temporal order of the dates of diagnosis to determine temporal correspondence (i.e. that the effect occurs after the cause); finally, we used GPT-4 to confirm or negate the relations we extracted due to the success that LLMs have shown in the literature for identifying causal relations (Arsenyan *et al.* 2024, Jiralerspong *et al.* 2024).

We have developed a DAG using the mined relations. We achieve this by defining scores that assess the strength of these relationships and transforming them into a structure similar to a SCM. While the score weighs each of the measures we define equally, a weighted combination of the scores can also be considered for ranking and removing edges from the graph. Generating a DAG enables us to employ techniques like the do-calculus (Pearl 2009) and build PRSs (Dudbridge 2016), demonstrating the graph’s effectiveness even if it does not exclusively feature strong causal relations. It is important to note that relations with low scores can still be plausible causal relations. For instance, it is well established that diabetes can lead to spontaneous abortion (Yilmaz *et al.* 2022). However, in UKB, the ϕ coefficient between spontaneous abortion (O03) and diabetes (E08–E13 and O24) is slightly negative (ϕ≈−0.006). This effect is plausible when we consider screening programs as possible confounders. In fact, the guidelines of the UK National Institute for Health and Care Excellence state that pregnant women at high risk of diabetes should be offered tests for gestational diabetes (National Institute for Health and Care Excellence 2015). Testing enables clinical interventions that are well-established to improve pregnancy outcomes (Saravanan *et al.* 2020).

We have illustrated how the DAG could be used to estimate the effect of therapeutic intervention in a precursor disease on an outcome disease using do-calculus. Furthermore, we demonstrated the potential of the generated DAG to compute PRSs. For instance, diseases such as diabetes and hypertension are commonly utilized as covariates in the risk scores of cardiovascular diseases (O’Sullivan *et al.* 2022). Moreover, studies on PRSs fitted using multiple traits have been shown to enhance PRS performance (Zhai *et al.* 2023). We showed that using an established PRS for a precursor disease to modify that of an outcome disease can increase the ability to estimate risk for the outcome condition in individuals. Additionally, we showed that the PRSs of precursor diseases could be used to generate a PRS for an outcome disease for which no target data was available. In the case where the PRS of an outcome disease is improved by our approach, we suggest that this may either be because there is an aggregation of contributory variants, i.e. more variants are used to determine the outcome disease’s PRS, or that contributing variants that were not sufficiently prevalent in the base data for the outcome disease’s PRS provide a significant signal in the contributing disorders.

We showed another application of the generated DAG in disentangling effects of variants. We were able to explain the effects that some variants have on an outcome disease through a link from one of its causative diseases according to our DAG. This work is in line with previous research which utilizes conditional independence and Mendelian randomization for mediation analysis of genetic variants (Hemani *et al.* 2017). By using our DAG, the mediation analysis can be automated for many variants and cause–outcome disease pairs on a large scale.

The relations in the causal network, or the DAG, we have generated can be directly integrated into computational methods and linked to knowledge graphs by means of the ICD-10-CM identifiers, forming a causal knowledge graph (Jaimini and Sheth 2022). In these graphs, diseases or phenotypes are represented by nodes, and edges are directed and potentially labeled or weighted. These knowledge graphs can be used for query answering and deductive inference as well as for causal inference. Representing disease–disease causality in a knowledge graph further permits linking of diseases to other kinds of biological entities through Linked Data principles (Bizer *et al.* 2023). Here, one limitation is our use of ICD-10-CM which is not used widely outside a clinical environment; however, the ICD-10-CM identifiers can be mapped to disease ontologies or phenotypes through the UMLS (Bodenreider 2004) or direct mappings included in disease ontologies (Vasilevsky *et al.* 2022, Schriml *et al.* 2022).

In contrast to studies that can handle risk scores for quantitative traits (Georgantas *et al.* 2024), our work is limited to binary outcomes of diseases. In the future, it may be possible to extend our work to systematically include quantitative traits. However, to include quantitative phenotypic traits, our text mining approach would need to be modified, in particular with respect to named entity recognition and normalization, and additional evaluation datasets and metrics would be required. As the data we generated is freely available, we expect further work in incorporating causal relations into biomedical knowledge graphs and ontologies, including those that capture quantitative traits. Furthermore, recent advances like Retrieval Augmented Generation (RAG) of LLMs may further enhance the extraction of relations from the literature (Borgeaud *et al.* 2022).

## 5 Conclusion

Our work provides a resource for causal relations between diseases as represented by ICD-10-CM, and each causal relation is supported by multiple sources of data. These causal relations can be compiled into a Directed Acyclic Graph (DAG) to generate a computable representation of the pathobiological relationships between diseases. The resulting graph can be used, for example, in generating PRSs, support generation of derived PRSs for diseases with no existing base data, or resolve pleiotropic effects of variants. Our causative data can therefore provide a link between genetic risk and the underlying pathophysiology of disease which can in turn be exploited to probe disease mechanisms and inform novel therapeutic approaches.

## Supplementary Material

btae639_Supplementary_Data

## Data Availability

The data including the disease dictionary, the full network, the DAG, the curated relations, and the PRSs are all available through https://github.com/bio-ontology-research-group/causal-relations-between-diseases. The DAG is also available via Zenodo https://zenodo.org/records/11368599.
