# Supplementary material for: Causal relationships between diseases mined from the literature improve the use of polygenic risk scores
Source: Bioinformatics. 2024 Oct 26;40(11):btae639. doi: 10.1093/bioinformatics/btae639 (PMC11639291; doi:10.1093/bioinformatics/btae639)
Supplement: btae639_Supplementary_Data [file btae639_supplementary_data.zip › 1.pdf]

Supplementary materials: Causal relationships between diseases mined from the literature improve the use of polygenic risk scores

Relation metric	Evaluation on curated samples (ROC AUC)
Phi correlation coefficient	0.79
Dependence	0.74
Date of diagnosis ratio	0.69
Number of annotations	0.87
GPT-4	0.68

Table 1: Evaluation of metrics against 100 expert-curated relations where 50 are positive and 50 are negative.

Causes	Outcome	Logistic regression
Hypertension, CHD	Cardiomegaly	0.581
Hypertension, CHD	Pulmonary edema	0.562

Table 2: ROC AUC results of diseases with no PRS. **Logistic regression** refers to the modified PRS fitted using logistic regression.

variant	p-value	mapped genes from GWAS Catalog
rs11591147	0.5484	PCSK9
rs11206803	0.2088	RPSAP20 - LINC01767
rs17114036	0.7099	PLPP3
rs646776	0.08262	CELSR2 - PSRC1
rs12133641	0.8805	IL6R
rs16986953	0.1093	LINC01808 - CISD1P1
rs16998073	0.3552	PRDM8 - FGF5
rs6841473	0.262	EDNRA
rs2306556	0.4559	GUCY1A1
rs72689147	0.6117	GUCY1A1
rs9349379	0.3949	PHACTR1
rs3130283	0.8672	PPT2-EGFL8, AGPAT1
rs78707197	0.06083	TENT5A
rs2327429	0.2266	TARID
rs2107595	0.5436	HDAC9 - TWIST1
rs57301765	0.3261	HDAC9 - TWIST1
rs9720071	0.1121	RN7SKP280 - INSIG1-DT
rs1926032	0.05128	CNNM2
rs73392700	0.1207	SIRT3
rs61904693	0.4745	DYNC2H1 - MIR4693
rs2128739	0.07038	DYNC2H1 - MIR4693
rs2839812	0.07038	DYNC2H1 - MIR4693
rs11226029	0.08928	DYNC2H1 - MIR4693
rs9515203	0.222	COL4A2
rs7183988	0.3255	FES
rs7500448	0.05331	CDH13
rs2738464	0.214	LDLR, SPC24
rs429358	0.7754	APOE
rs7412	0.4643	APOE
rs28451064	0.3502	LINC00310 - KCNE2

Table 3: Conditionally independent variants

Patterns	
attributed to	attributable to
cause	causes
caused by	because of
lead to	led to
leads to	owing to
secondary to	due to
result in	resulting from
arising from	results in
produced by	induced by

Table 4: Used lexical patterns between diseases

Prompt
I will give you a list of directed pairs of ICD-10 codes, tell me if the first can cause the second to occur, answer yes or no:<br>1. K40 – Q53.9<br>2. D17.9 – G56.3<br>3. B34.9 – N08<br>4. M30.0 – K76.9<br>5. E74.4 – E87.20

Table 5: Prompt example

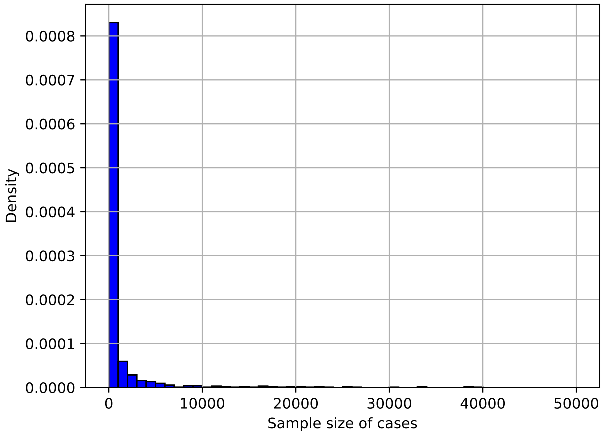

Figure 1: Distribution of the sample size of disease cases in the UKB. The density represents the proportion of data points within each bin, normalized such that the total area under the histogram equals 1.

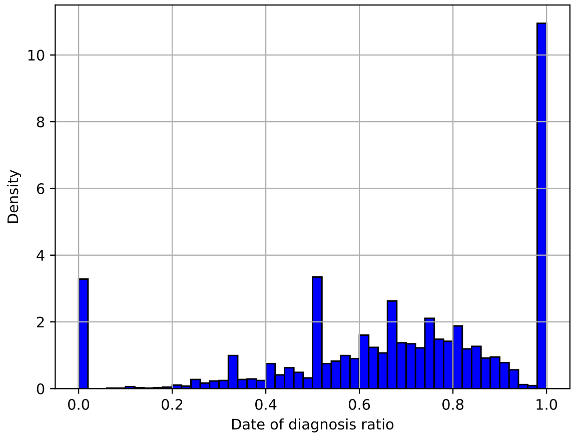

Figure 2: Distribution of the date of diagnosis ratio. The density represents the proportion of data points within each bin, normalized such that the total area under the histogram equals 1.

Expert curation strategy

Disease pairs or causal chains were initially reviewed by a domain expert (P.N. Schofield) and literature searches carried out for recent reviews on the pathology, epidemiology and genetics of the diseases. In many cases, the relationships between diseases have been well established for some time and are covered by professional medical texts. In other cases, the review, guided by the knowledge of the expert, were assessed on the basis of some of the Bradford Hill criteria:

- A strong statistical correlation between the suggested causative and consequent diseases.
- Repeatability, especially when studies are orthogonal, so where there are independent studies of different groups showing concordant conclusions.
- Directional temporal dependence of one disease on another.
- Plausibility of a pathophysiological mechanism linking one disease to its dependent disease.

There were no data on the effects of drugs or environmental agents in this study, and so this criterion was not used. Because the available information on each disease pair is highly variable, the combination of strong correlation, multiple concordant studies and temporal dependence was taken as evidence for a causal link. The judgement of the evidence and the strength of the evidence was made on the basis of the expertise of the domain expert.

The Bradford Hill criteria, based on multiple orthogonal sources of information, are widely used in an epidemiological context in evidence-based medicine. They include consideration of the strengths of association between variables, reproducibility, dose-response relationships (where applicable), temporal sequencing and plausible mechanisms [6, 4].

Expert validation of the case study

Expert validation of the causal relationships between $APOE$, $PCSK9$ variants, Coronary heart disease and angina pectoris

We established causal hypotheses for variants in $APOE$ and $PCSK9$ causing coronary heart disease (CHD) and angina pectoris (AP). Evidence consistent with the Bradford Hill criteria is available for this causative chain. Variants in the $APOE$ and $PCSK9$ genes are strongly statistically related to coronary heart disease. $APOE$ is established as a major contributor to atherosclerosis through its role in lipid transport and metabolism, and mechanistically through dyslipidemia leading to coronary atherosclerosis and thence to CHD. Recent studies also suggest that some affects may be mediated through a role in inflammatory processes. Lines of supporting evidence are wide-ranging including: prospective epidemiological studies, Mendelian randomisation studies, physiological observations and associations, animal experimentation, mutagenesis, and therapeutic interventions [8, 10, 5, 11, 9]. $PCSK9$ is a key gene in the regulation of cholesterol metabolism and plasma lipoprotein levels. Inherited variants are associated with familial hypercholesterolemia and sporadic variants with hyper- and hypo-cholesterolemia and CHD, with mechanisms converging with those of $APOE$ via coronary atherosclerosis and CHD [7]. Coronary atherosclerosis is a cause of AP. There are several sub-types of AP, reviewed by [1, 12], but we are unable to distinguish between these due to the granularity of the ICD-10-CM term used. Most commonly, stable angina is caused, in CHD, by reduced cardiac perfusion, for example by occlusion or inadequate dilation of the coronary vessels, or defects in the microcirculation (see references in [1]) as a consequence of coronary atherosclerosis. We can consequently establish evidence for a causal relation through statistical association, temporality (prospective studies), mechanistic studies in model organisms and cell culture, interventional effects, dose relationships (severity of lipid dysregulation/severity

of atherosclerosis/risk of disease onset). We conclude that the causal chain established by our method, and its linking to causal genetic variants through PRS, is plausible.

Automatic polygenic score calculation

We first excluded samples with high heterozygosity or genotype missing rates and discrepancies between reported and genetic sex. We also filtered out first-degree relatives based on the kinship coefficient provided by UKB (data field 22012). We further and restricted our analysis to white British individuals which are the majority in UKB, resulting in 425,573 individuals. We created a random split of the filtered samples where 70% were used to fit the Genome Wide Associate Studies (GWASs), 10% for the fitting of the PRSs (including the modified PRSs), and 20% for testing.

We conducted GWAS analysis using Plink [2], applying several quality control filters. Specifically, we set a Hardy-Weinberg equilibrium (p-value threshold of 1×10^{-6}) to exclude single nucleotide polymorphisms (SNPs) that deviate significantly from expected genotype frequencies. We also excluded individuals with more than 10% missing genotype data, which helps maintain the integrity of the sample population. Furthermore, we filtered SNPs for missing genotype data, removing those with more than 10% missing calls. Finally, we applied a minor allele frequency threshold of 0.01 to exclude rare variants. We fitted the risk scores using PRSice [3] with the default parameters and the GWASs conducted earlier to find the best fit PRS on the 10% split of data. The testing was carried out on the remaning 20% split of the data.

References

[1] Cristina Balla, and Rita Pavasini, and Roberto Ferrari. Treatment of angina: Where are we? *Cardiology*, 140(1):52–67, 2018.

[2] Christopher C Chang et al. The second generation of the PLINK software for genotype data. *GigaScience*, 4(1):1–4, 2015.

[3] Shing Wan Choi and Paul F O'Reilly. PRSice-2: Polygenic Risk Score software for biobank-scale data. *GigaScience*, 8(7):giz082, 2019.

[4] Kristen M. Fedak et al. Applying the bradford hill criteria in the 21st century: how data integration has changed causal inference in molecular epidemiology. *Emerging Themes in Epidemiology*, 12(1), September 2015.

[5] Marit Granér et al. Apolipoprotein e polymorphism is associated with both carotid and coronary atherosclerosis in patients with coronary artery disease. *Nutrition, Metabolism and Cardiovascular Diseases*, 18(4):271–277, May 2008.

[6] Austin Bradford Hill. The environment and disease: Association or causation? *Proceedings of the Royal Society of Medicine*, 58(5):295–300, May 1965.

[7] Sandra Hummelgaard et al. Targeting pcsk9 to tackle cardiovascular disease. *Pharmacology amp; Therapeutics*, 249:108480, September 2023.

[8] Peter Libby et al. Atherosclerosis. *Nature Reviews Disease Primers*, 5(1):56, August 2019.

[9] Matthew W. McMaster et al. The impact of the apolipoprotein e genotype on cardiovascular disease and cognitive disorders. *Cardiology in Review. E.Pub in Press*, April 2024.

[10] Børge G. Nordestgaard. Triglyceride-rich lipoproteins and atherosclerotic cardiovascular disease: New insights from epidemiology, genetics, and biology. *Circulation Research*, 118(4):547–563, February 2016.

[11] Michael J. Pencina et al. Predicting the 30-year risk of cardiovascular disease: The framingham heart study. *Circulation*, 119(24):3078–3084, June 2009.

[12] Zeev Vlodaver, and Henry N Neufeld, and Jesse E Edwards. Pathology of angina pectoris. *Circulation*, 46(6):1048–1064, 1972.
